# Impact of weight reduction surgery on static and dynamic lung volumes

**DOI:** 10.1016/j.amsu.2021.102457

**Published:** 2021-06-01

**Authors:** Ibrahim Falih Noori, Azza Sajid Jabbar

**Affiliations:** a Department of Surgery, College of Medicine, University of Basrah, Iraq; b Department of Toxicology, College of Pharmacy, University of Basrah, Iraq

**Keywords:** Morbid obesity, Pulmonary volumes, Function tests, Weight reduction surgery

## Abstract

**Background:**

Obesity could affect many functions of the body systems, particularly respiratory system. Effect of obesity on respiratory system leads to an impairment in pulmonary function tests which is represented by a decrease in lung volumes and capacities, therefore obstructive or restrictive pulmonary diseases may develop. The recent study was conducted to investigate and assess the impact of weight loss by surgery on static and dynamic lung volumes (pulmonary function tests) and the improvement in co morbidities.

**Patients and methods:**

The study included 68 morbid obese patients, 36 females and 32 males. The patients were with age range 24–56 years, BMI≥ 40 kg/m^2^or≥35 kg/m^2^ with co morbidities. Pulmonary volumes and function tests of all patients were measured before weight loss surgery and one year after the surgery.

**Result:**

The results showed a significant reduction in the body weight (p < 0.05), with an improvement in co morbidities. Pulmonary volumes ERV,IRV,TLC, FRC and RV were significantly changed one year after surgery as well as there were significant increases in the mean values of the dynamic volumes such as FEV1,FEV1%,FEF50%, PEF and MVV.(p < 0.05).

**Conclusion:**

loss of excess body weight by bariatric surgery resulted in a significant improvement in co morbidities and function of respiratory system represented by significant changes in both static and dynamic lung volumes …

## Highlights

• Morbid obesity leads to an impairment in pulmonary functions represented by decrease in lung volumes and capacities.
• Dynamic pulmonary volumes such as ERV, IRV, TLC, FRC & RV and static volumes such as FEV1, FEV1%, PEF & MVV were significantly improved one year after surgery.
• Bariatric surgery results in significant improvement of both obstructive and restrictive pulmonary diseases.

## 1 Introduction

Morbid obesity has become a world-wide health problem and reached an epidemic level in several societies. A morbidly obese individual is one with body mass index(BMI) (ratio of individual height to weight about 40 and more or the ratio is 35 and more with co-morbidities and obesity related problems such as arterial hypertension, diabetes,cardiovascular diseases and hyperlipidemia. An individual is also considered morbidly obese when the body weight is 100 pounds over the ideal body weight [1,2].

The well-known health problems associated with morbid obesity include type 2 diabetes ischemic heart diseases osteoarthritis, GERD, depression, infertility, respiratory problems and sleep apnea. It is thought that the overall effects of obesity on pulmonary function are multi-factorial that is related to mechanical and inflammatory effect of abnormal deposition and accumulation of fatty tissues [3,4].

According to the WHO report in 2008 that more than one billion are overweight and more than 300–500 millions are morbidly obese individual that have marked limitation in overall physical activity including respiratory function [5]. Obesity can result in compromise in lung function represented by a decrease in pulmonary volumes, a decrease in the strength of respiratory muscles and consequently increase in respiratory work and limitation and restriction in diaphragm movement, leading to hypoventilation and hypoxemia manifested by dispend with rapid and shallow breathing [6,7]. The effect of obesity on pulmonary function therefore, is mainly caused by interfering with ventilator mechanics as well as inflammatory factor that adversely affect pulmonary function. It has been well documented that the main changes in respiratory functions related to morbid obesity is consequently a decrease in lung function and volumes represented by decreased expiratory reserve volume (ERV) and functional residual capacity (FRC) associated with restrictive type of respiration [8,9]. Weight reduction surgery nowadays is the only efficient modality that results in a sustained and significant weight loss with valid improvement in obesity related co-morbidities, quality of life and mortality [10].

The aim of the present study was to investigate the role of weight loss by surgery in the improvement of respiratory function by measuring static and dynamic lung volumes (pulmonary function tests) of morbidly obese patients before weight loss surgery and one year after the surgery, beside its role in improving the patient's co morbidities.

## 2 Methods

This is a prospective controlled study, conducted for the period between Sept.2017 and April 2019) which included 68 morbid obese patients (36 females and 32 males) with age range 24–56 years and BMI more than 40 kg/m^2^ or 35 kg/m^2^ with co-morbidities. These were presented for weight reduction surgery in form of sleeve gastrostomy at major hospitals in Basra City. Static and dynamic lung volumes of each patient were measured twice: first measurement was before the surgery and the second measurement was one year after surgery. The study was approved by the local ethic committee, and signed informed consents were obtained from all participants.

Demographics and characteristic of all patients including: age, sex, height, weight, BMI co-morbidities, drug taken and smoking status were obtained by a questionnaire.Patients enrolled in this study were measured prior to investigate pulmonary function tests for any chronic obstructive air way diseases (COPD), chronic restrictive airway diseases (CRPD)and neuromuscular disorders in order to exclude any abnormality and disorder. Dispend when present was assessed using the modified medical research council skill (marc) which is the most commonly and widely used skill to assess the grade of dispend. Lung volumes measurement was conducted using Micro Medical Spiro meter III (MIR Spiro lab III Diagnostic Spirometer, Ltd.England).Measurement of pulmonary function tests was performed by a single well trained physician for the patient in a sitting position with nose clip in place in a room of 22–25 C° temperature to minimize the difference between the temperature of spirometer and body temperature. The mouth piece is applied and fitted well to prevent any leak with emphasis on the full inspiration followed by rapid and sustained exhalation until the procedure is stopped. All the measurements of pulmonary function tests and volumes were done before 12:00 p.m. The parameters that were measured include static lung volumes:ERV,IRV,TLC, FRC and RV and dynamic lung volumes(pulmonary function tests) such as FVC,FEV1, FEV%, FEF50%, PEFand MVV. At least three complete curves and a maximum three measures were recorded and adopted value was taken. All the procedures were done according to the American thoracic society guidelines. The results were analyzed and compared to show the changes in the lung parameters among morbidly obese patients prior and one year after bariatric surgery. The study is work is reported in line with the CONSORT criteria and was registered at Research registry: http://www.researchregistry.com. Registration ID: researchregistry 6797. Hyperlink: https://www.researchregistry.com/browse-the-registry#home/

### 2.1 Statistical analysis

Statistical analysis was carried out using Statistical Package for the Social Sciences (SPSS) Statistical Software for Windows, Version 24.0 IBM (SPSS Inc, IL, and USA). The data are represented as means value ± standard deviation (SD). Independent student-test was used to compare between the mean values of the two measurements. Pearson correlation test was used to find the correlation between lung parameters and bodyweight parameters. The result was considered significant at p < 0.05.

## 3 Results

Sixty eight morbidly obese patients presented for sleeve mastectomy as a type of weight reduction surgery. These patients were 36 (52.9%) female patients and 32 (47.1%) male patients. The preoperative baseline characteristic of these patients including age, weight, height, MBI and co-morbidities are summarized in (Table 1). The overall mean of the patient's age was 41.9 ± 5.3 (43.1 ± 7.1 for male versus 39.6 ± 6.5 for females). The preoperative of the body weight was 128.45 ± 9.8 (range 96–182 kg) and mean BMI was 52.35 ± 3.8 kg/m^2^ (range 42.6–62.1 kg/m^2^).The results showed a significant reduction in mean body weight and BMI one year after surgery (79.4 ± 8.2 and 28.32 ± 2.1) respectively,p < 0.05, as seen in Table 2.

**Table 1:** The preoperative baseline characteristics of the patients.

Variable sex (No)	NO. (%)	Age means	Weight(kg) Means	BMI Means	Smoking status NO. (%0)	Co-morbidity^a^ NO. (%)
Males	32 (47.1%)	28-56 (43.1 ± 7.1)	94-178 (131.1 ± 6.7)	48.9 ± 1.4	9 (13.2%)	13 (19.1%)
Females	36 (52.9%)	24-52 (39.6 ± 6.5)	103-184 (126.6 ± 4.11)	53.4 ± 2.5	0	18 (26.47%)
Total	68	24-56 (41.9 ± 5.3)	94-184 (128.45 ± 9.8)	52.35 ± 3.8	9 (13.2%)	31 (45.58%)

a The main co-morbidities investigated in this study were Diabetes mellitus, Arterial hypertension & hyperlipidemia and their concurrent medications.

**Table 2:** The differences in body weight, BMI and co morbidities between preoperative and postoperative patients groups.

Patients	Weight(kg) mean ± SD	BMI mean ± SD	Co-morbidity NO.
Preoperative	128.45 ± 9.83	52.35 ± 3.8	31 (45.58%)
			(13 male+18 female)
Postoperative	79.4 ± 8.2	28.32 ± 2.1	14(20.58%)
			(9 males +5 females)
*P value	0.0021	0.0051	0.0001

*P is significant at level> 0.05.

Before weight loss surgery out of 68 patients thirty one patients (45.58%) were with comorbidities 13 (19.1%) males and 18(26.47%)females, as seen in Table 2. Distribution of the morbidities among the male patients was 7 hypertensive,4 diabetic and 2 dyslipidemia. While in female, the distribution was 9 hypertensive, 6 diabetic and 3 dyslipidemia. All these patients were on regular and continuous medications, with no evidence of presence of COPD. On the other hand, one year after surgery the postoperative patients showed a reduction in the percentage of co morbidities to 20.52% (Table 2).In general fourteen patients remained with comorbidities, 5 males(3 hypertensive and 2 diabetics)and 9 females(6 hypertensive and 3 diabetics).No one was with dyslipidemia one year after surgery, as seen in Table 2. Those with persistent co-morbidity were less sever (mild to moderate and) and easily controlled than before surgery with decreased doses of medications.

Table 3 showed that 70–100% of excess body weight was lost in 25 patients (36.76%), 50–70% of excess body weight in 19 patients (27.94%).Body weight of 14 patients (20.58%)and 10 patients (14.72%) were decreased to the range of overweight one year after surgery, although there was a significant decreased in the their body weight and their BMI. Therefore the mean of BMI was significantly decreased one year after surgery.

**Table 3:** Percentage of excess body weight loss in postoperative patients.

Percentage of weight loss	NO. Of patients (%)	BMI Means
70–100	25 (36.76)	25.1
50–70	19 (27.94)	25.6
50–25	14 (20.58)	30.6
≤25	10 (14.72)	32.0

**Static lung volumes:** Data analysis showed that the lung volumes and capacities significantly changed in postoperative patients one year after surgery.These changes were either a significant increase as in ERV and TLC (1.18 ± 0.34 and 117 ± 12.2), p > 0.05 or a significant decrease as in IRV, FRC and RV (1.42 ± 0.33, 89.2 ± 7.41 and 76 ± 6.23) respectively, p > 0.05, as seen in Table 4.

**Table 4:** A preoperative and postoperative (one year after weight reduction surgery) comparison of static lung volumes indices.

Lung volumes (L)	Preoperative*	One year* postoperative	P value
ERV(L)	0.51 ± 0.26	1.18 ± 0.34	0.0053
IRV(L)	1.79 ± 0.51	1.42 ± 0.33	0.0082
TLC(L)	109 ± 16.31	117 ± 12.2	0.0068
FRC(L)	111.2 ± 7.52	89.2 ± 7.41	0.0072
RV(L)	98 ± 2.3	76 ± 6.23	0.0029

*Data are represented by mean ± SD.

**P is significant statistics at level< 0.05.

***ERV: Expiratory Reserve Volume, IRV: Inspiratory Reserve Volume, TLC: Total Lung Capacity, FRC: Functional Residual Capacity, RV: Residual Volume.

**Dynamic lung volumes (Pulmonary function tests)**The results showed that there were significant increases in the pulmonary function tests of postoperative patients in one year after bariatric surgery,p > 0.05, as seen in Table 5.The increases in the studied parameters referred to an improvement of respiratory airflow and respiratory endurance.

**Table 5:** A preoperative (baseline) and postoperative (one year after weight reduction surgery) comparison of dynamic lung volumes indices.

Pulmonary function tests	Preoperative*	One year* postoperative	P value
FVC(L)	3.8 ± 1.12	4.62 ± 0.89	0.013
FEV1(L)	3.1 ± 0.12	3.92 ± 0.16	0.002
FEV1%	81.91 ± 9.8	90.71 ± 3.31	0.0065
FEF50%	1.82 ± 0.71	3.37 ± 0.93	0.0011
PEF(L/S)	4.23 ± 1.72	7.57 ± 1.82	0.0021
MVV(L/S)	11.02 ± 1.68	13.98 ± 1.52	0.036

*Data are represented by mean ± SD.

**P is significant statistically at level< 0.05.

**FVC: Forced Vital Capacity, FEV1: Forced Expiratory Volume at the first second of expiration, FEV1%: Percentage of FEV1, FEF50%: Forced Expiratory Flow at 50% of vital capacity, PEF: Peak Expiratory Flow, MVV: Maximum Voluntary Ventilation.

**Correlation between pulmonary function tests parameters and body weight parameters (weight and BMI).**The results showed that there were significant negative correlations between bodyweight and each of FVC, PEF and MVV, p > 0.05, as seen in Table 6. The same finding was reported to the correlation between each of these pulmonary function tests and BMI, P > 0.05, in a way that confirms the adverse correlation between pulmonary function and weight reduction by surgery, as seen in the same table.

**Table 6:** Correlation between body weight parameters (weight&MBI) and pulmonary function tests.

Variable	Body weight r p		BMI r p	
FVC	−0.382	0.021	-.0.385	0.033
PEF	−0.427	0.0041	−0.542	0.0037
MVV	−0.378	0.013	−0.409	0.011

Correlation is considered significant at level <0.05 (2-tailed).

## 4 Discussion

Obesity can cause many systemic complications which result in sever and even permanent impairment of many organs functions.It leads to diminish the life quality and elevate the risk of serious diseases such as diabetes mellitus,hypertension and dyslipidemia [8,11].Therefore, as we found in this study, the preoperative obese patients showed greater prevalence of co morbidities compared to the postoperative group, as shown in Table 2. The reduction in the mean of bodyweight was from 128.45 ± 9.83 to 79.4 ± 8.2 and the change in BMI was from 52.35 ± 3.8 to 28.32 ± 2.1 (p > 0.05) one year after weight loss surgery. The decline in the body weight and BMI resulted in a reduction in co morbidities from 31 patients (45.58%) to 14 patients (20.58%).The adverse effect of obesity is due to many reasons related to excessive and progressive accumulation of adipose tissue in the organs in a way that affects their functions, in addition to the metabolic changes and increased inflammatory processes caused by elevated level of numerous cytokines that produced by adipose tissues [12].Other factor that may contribute to develop the co morbidities in obese patients is formation of reactive oxygen species [13].However an accumulation of fat in the body organs and systems results in various changes in their physiology [14].In the recent study, most of patients have lost weight one year after bariatric surgery. The reduction in the body weight was in different percentage as seen in Table 3,70–100% of the body weight was lost in 27 patients (39.7%) and 21 patients (30.88%) have lost 50–70% of their weight, hence there was significant reduction in the number of co morbidities, mainly type 2 diabetes mellitus and arterial hypertension. The role of weight loss surgery has been proven by previous clinical studies [8,15,16].

Beside the significant reduction in weight and BMI, which expected in such type of surgery, our results showed significant improvement in lung volumes and capacities (Table 4). This improvement was represented by significant increases in each of ERV and TLC (p > 0.05). This result was in agreement with the results of other studies [15,16].Obesity results in mechanical compression of lung, diaphragm and chest cavity leading to a restrictive lung impairment, a decrease in total pulmonary compliance, an increase in pulmonary resistance as well as a decrease in the respiratory muscles strength [17].Furthermore deposition of intra-abdominal fat results in splinting of the diaphragm. All these abnormalities lead to increase work of breathing and decrease in lung volumes [18,19]. An improvement in lung volumes was also reported by Thomas PS et al. [20] who showed in their prospective study of the changes in lung volume, carbon transfer and arterial blood gas tension undertaken in 29 morbidly obese patients before and after bariatric surgery, that weight reduction surgery resulted in significant improvement in lung volumes. Most changes in TLC of morbid obese patients is accounted for by a change in ERV and RV revealing that splinting of diaphragm by abnormal deposition of intra-abdominal fat prevents its full movement and expansion. This finding was also emphasized by other studies [6,21], which stated that the decrease in the in ERV is a well-known changes in respiratory function caused by morbid obesity, which is explained by restriction of diaphragm movement due to upward pressure by distended and increased abdominal cavity of morbidly obese patients. Previous studies [16,22] indicated to an improvement in ERV and lung capacity after weight loss surgery. The increase in lung volumes due to weight loss were also reported by Young et al. [23] and Weiner et at [24]. In their comparative studies of pulmonary functions tests between obese and non obese sedentary female patients. Furthermore, we found significant reductions in IRV, FRC and RV, (p > 0.05) which indicated an improvement in the respiratory muscles performance and reduction in the pulmonary resistance. This finding was similar to the result of other study by Costa et al. [25], which studied the influence of excessive weight loss, with decreased muscle mass, on pulmonary function was evaluated in 16 obese patients. They conclude that lung function tests showed significant increase of vital capacity, functional residual capacity, total lung capacity and maximal voluntary ventilation after significant weight loss. The changes in ERV and IRV of obese patients were attributed to the presence of problem in mechanics of the chest cavity. The reduction in the ERV is mainly due to increase in the respiratory drive and dyspnea, with significant improvement after bariatric surgery. These results could be explained by the fact that the decreased ERV due to obesity causes a respiratory overload that leads to increase in the respiratory drive and dyspnea [26].

When comparing pulmonary function tests between preoperative group and postoperative group, each of FEV1, FVC, FEV1%, FEF50%, PEF and MVV were significantly higher (p > 0.05) in postoperative one.These positive changes could be attributed to the improvement in the factors that resulted in a limitation on in the chest wall expansion and airflow after weight loss surgery, leading to reduce the work of breathing and improve the parameters that assess airflow and pulmonary endurance. These findings were similar to what reported by El-Gamal et al. [3] and Nguyen NT et al [27] in their prospective study of 28 patients to investigate the relationship between dyspnea, pulmonary function results, and respiratory drive in morbidly obese patients before and after weight loss., the results showed weight reduction surgery results in a significant reduction in BMI and respiratory drive measurements as well as significant improvement in dyspnea that attributed to the alteration in the chest mechanisms and that lung components have been rearranged inside the ribcage in a way that affects positively on the mechanisms of ventilation. Therefore each of static lung volumes and pulmonary function tests showed an improvement.

## 5 Conclusion

We conclude that weight reduction by surgery was significantly associated with the reduction of co morbidities such as diabetes mellitus, hypertension and dyslipidemia. Other important outcome is the positive role of weight reduction by surgery in the improvement of functional efficiency of respiratory system as evidenced by each of static lung volumes and capacities such as ERV, IRV,VC, FRC and RV and dynamic lung volumes which were represented by FEV1, FVC,FEV1%, PEF and MVV. The positive role of weight reduction by surgery on the function of respiratory system was ensure by the inverse correlation between the studied lung volumes and body weight, even though more studies with extra respiratory and ventilator markers as well as more patients number are required in order to confirm this outcome.

## Ethical approval

The study was approved by ethical committee of Basra medical college, University of Basra.

## Sources of funding

None, Self-funded.

## Author contribution

The surgical aspect of the study was written by dr. Ibrahim Falih Noori and the physiological aspect and changes in respiratory function tests was written by dr.azza sajid jabbar.

## Research registration number

Name of the registry: www.researchregistry.com.

Unique identifying number or registration ID: researchregistry **6797.**

**Hyperlink:** https://www.researchregistry.com/browse-the-registry#home/.

## Guarantor

The authors are the sole guarantors for this work.

## Consent

Written informed consent was obtained from all the patients for publication of this case series.

## Provenance and peer review

Not commissioned, externally peer-reviewed.

## Declaration of competing interest

The authors declare no any conflicts of interest.
